# 

**Published:** 1885-07

**Authors:** 


PAGE.

ORIGINAL COMMUNICATIONS.

Hay Fever: Its Cause and Cure. E. F. Ingals, M.D. .... 1
The Continued Pelvic Curve in the Obstetric Forceps, etc. E. W. Sawyer, M.D. .... 15
The Normal Position of the Uterus, etc. F. H. Martin, M.D .... 23

EDITORIAL.

Proceedings of the American Medical Association's . Amended Committee relative to the next International Medical Congress .... 37
Inoculation for Cholera and Yellow Fever .... 40
Atheroma of the Coronary Arteries and its Relations to Certain Cases of Cardiac Failure .... 41

SOCIETY PROCEEDINGS.

Chicago Medical Society. Meeting June 15th. A Successful Case of Nephrectomy. R. G. Bogue, M.D. 45
Meeting July 6th. Exhibition and Explanation of Microscopical Specimens of Aspergillus Glaucus. Osteoma of a Canine Tooth. Mycelia from a Tonsil. R. Tilley, M.D. .... 50
Treatment of Acute Coryza. J. A. Robinson, M.D .... 52
Chicago Gynecological Society, Meeting May 29th. Normal Position of the Uterus. F. H. Martin, M.D. .... 58
Michigan State Medical Society. Twentieth Annual Meeting, at Port Huron, June 10th .... 64

CLINICAL REPORTS.

Mercy Hospital, Chicago. Service of Professor E. Andrews. *Total Obstruction of the Colon for four months—Colotomy* .... 68

PAGE.

Carcinoma of the Pylorus. Laparotomy .... 69
Litholapaxy—Two Cases .... 70

EXTRACTS.

Uses of Traumaticine .... 71
Ecbolic Action of Pilocarpin .... 72

STATE BOARD OF HEALTH.

Transactions of the Illinois State Board of Health. Meeting April 16th .... 76

BOOK REVIEWS.

Modern Therapeutics of Diseases of Children. Edwards .... 93
Hand-Book of Diseases of the Skin. Ziemssen's Cyclopedia .... 95
Micro-Chemistry of Poisons. Wormley .... 99
On the Treatment of Gonorrhœa. Milton .... 101
Clinical Studies of Diseases of the Eye. Arlt .... 102
The Oleates. Shoemaker .... 103
Experimental Pharmacology. Hermann .... 104
A Text-Book of Practical Medicine. Loomis .... 105
Experimental Researches on Cicatrization in Blood-vessels after Ligature. Senn .... 105
A Manual of Dermatology. Robinson 107
Voice, Song and Speech. Browne. 110

NEWS ITEMS.

The Alumni Association Prize of the College of Physicians and Surgeons of New York .... 111
A New Medical and Surgical Directory of the United States .... 111

LOCAL NEWS .... 112

